# Supplementary material for: The Impact of Partial Splenic Embolization on Portal Hypertensive Gastropathy in Cirrhotic Patients with Portal Hypertension
Source: J Clin Med. 2023 Apr 3;12(7):2662. doi: 10.3390/jcm12072662 (PMC10094775; doi:10.3390/jcm12072662)
Supplement: Supplementary file 1 [file jcm-12-02662-s001.zip › jcm-2300817-supplementary.pdf]

Table S1. Efficacy of partial splenic embolization on portal hypertensive gastropathy based on Child–Pugh score.

	**Improved group**	**Non-improved group**	**Amelioration rate (%)**
Child–Pugh score 5	5	1	83
Child–Pugh score 6	5	1	83
Child–Pugh score 7	5	5	50
Child–Pugh score 8	3	3	50
Child–Pugh score 9	0	3	0
